# Validation in Zambia of a cervical screening strategy including HPV genotyping and artificial intelligence (AI)-based automated visual evaluation

**DOI:** 10.1186/s13027-023-00536-5

**Published:** 2023-10-16

**Authors:** Groesbeck P. Parham, Didem Egemen, Brian Befano, Mulindi H. Mwanahamuntu, Ana Cecilia Rodriguez, Sameer Antani, Samson Chisele, Mukatimui Kalima Munalula, Friday Kaunga, Francis Musonda, Evans Malyangu, Aaron Lunda Shibemba, Silvia de Sanjose, Mark Schiffman, Vikrant V. Sahasrabuddhe

**Affiliations:** 1 Women and Newborn Hospital, University Teaching Hospital, Lusaka, Zambia; 2 HPV-AVE (PAVE) Initiative, Division of Cancer Epidemiology and Genetics, National Cancer Institute, Rockville, MD 20850 USA; 3 Information Management Services Inc, Calverton, MD USA; 4 National Library of Medicine, NIH, Bethesda, MD 20892 USA; 5 Division of Cancer Prevention, National Cancer Institute, Rockville, MD 20850 USA

**Keywords:** Cervical cancer screening, HPV typing, AI, Risk-based management

## Abstract

**Background:**

WHO has recommended HPV testing for cervical screening where it is practical and affordable. If used, it is important to both clarify and implement the clinical management of positive results. We estimated the performance in Lusaka, Zambia of a novel screening/triage approach combining HPV typing with visual assessment assisted by a deep-learning approach called automated visual evaluation (AVE).

**Methods:**

In this well-established cervical cancer screening program nested inside public sector primary care health facilities, experienced nurses examined women with high-quality digital cameras; the magnified illuminated images permit inspection of the surface morphology of the cervix and expert telemedicine quality assurance. Emphasizing sensitive criteria to avoid missing precancer/cancer, ~ 25% of women screen positive, reflecting partly the high HIV prevalence. Visual screen-positive women are treated in the same visit by trained nurses using either ablation (~ 60%) or LLETZ excision, or referred for LLETZ or more extensive surgery as needed. We added research elements (which did not influence clinical care) including collection of HPV specimens for testing and typing with BD Onclarity™ with a five channel output (HPV16, HPV18/45, HPV31/33/52/58, HPV35/39/51/56/59/66/68, human DNA control), and collection of triplicate cervical images with a Samsung Galaxy J8 smartphone camera™ that were analyzed using AVE, an AI-based algorithm pre-trained on a large NCI cervical image archive. The four HPV groups and three AVE classes were crossed to create a 12-level risk scale, ranking participants in order of predicted risk of precancer. We evaluated the risk scale and assessed how well it predicted the observed diagnosis of precancer/cancer.

**Results:**

HPV type, AVE classification, and the 12-level risk scale all were strongly associated with degree of histologic outcome. The AVE classification showed good reproducibility between replicates, and added finer predictive accuracy to each HPV type group. Women living with HIV had higher prevalence of precancer/cancer; the HPV-AVE risk categories strongly predicted diagnostic findings in these women as well.

**Conclusions:**

These results support the theoretical efficacy of HPV-AVE-based risk estimation for cervical screening. If HPV testing can be made affordable, cost-effective and point of care, this risk-based approach could be one management option for HPV-positive women.

**Supplementary Information:**

The online version contains supplementary material available at 10.1186/s13027-023-00536-5.

## Background

In the ongoing initiative to eliminate cervical cancer, the World Health Organization (WHO) highlights the central role of HPV [1]. The recently demonstrated efficacy of single-dose HPV vaccination provides hope for eventual primary prevention of cervical cancer [2]. However, implementing affordable and accurate HPV screening is still a major challenge in lower-resource settings [3–6].

Where HPV testing is done, a negative HPV result in mid-adulthood provides strong reassurance that cervical cancer/precancer is not present or imminent [7, 8]. While the high negative predictive value is settled, feasibility of HPV testing remains an unsettled issue especially in lower-resource settings. A major practical issue is the clinical management of women testing HPV-positive [3]. WHO recommends either ablation/excision of the cervical transformation zone in its entirety in all women testing HPV-positive ("screen-treat") or performance of an additional test on HPV-positive women to determine who will benefit most from treatment ("screen-triage-treat") [1].

As a novel screen-triage-treat strategy, we are evaluating two complementary tests: HPV genotyping and artificial intelligence (AI)-assisted visual evaluation. Regarding HPV genotyping, there are approximately a dozen HPV types classified as carcinogenic, and the type of HPV strongly modifies risk of cancer, i.e., the risk groups in descending order of carcinogenicity are HPV16, then HPV18/45, then HPV31/33/35/52/58, then HPV39/51/56/59/68 [9, 10]. HPV typing could be provided at minimal added costs compared with HPV positivity/negativity, and several assays have incorporated HPV typing as part of their readout of results [5, 11].

The second complementary triage method is a visual adjunct called "automated visual evaluation (AVE)". AVE is a real-time, deep learning-based classifier of cervical appearance with a readout as either reflective of precancer/cancer, indeterminate, or normal based on images captured by a digital camera [12–15].

**Fig. 1:**
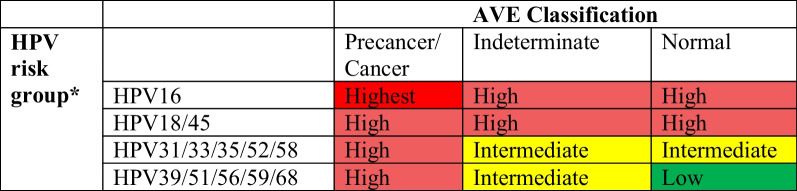
Combination of HPV typing and automated visual evaluation (AVE) to create risk score for cervical screening. *In case of multiple infections, the result will be hierarchical, as HPV16 positive, else (if HPV16 negative) HPV18/45 positive, else (if HPV16 and HPV18/45 negative) HPV31/33/35/52/58 positive, else (if HPV16 and HPV18/45 and HPV31/33/35/52/58 negative) HPV39/51/56/59/68 positive, else negative

The cross-combination of the four-level HPV type groups with the three-level AVE algorithm score creates 12 risk levels (Fig. 1), a gradient that could help clinicians identify which HPV-positive women are most likely to have precancer and are therefore in greatest need of ablation or excisional cervical treatments to prevent cervical cancer [3].

We present an evaluation of the HPV-AVE approach for predicting precancer/cancer based on screening results and histologic outcomes in the screening program in Lusaka, Zambia.

## Methods

### Study population and field study

The research took place in the well-established cervical cancer screening program nested inside public sector primary care health facilities in Lusaka [16, 17]. Women in this analysis were recruited under informed consent from clinics which primarily offer screening and treatment with thermal ablation or large loop excision of the transformation zone (LLETZ) by trained nurses. The standard of care screening was conducted by experienced nurses who examined women with visual inspection with acetic acid (VIA)-based screening aided with high-quality digital cameras; the magnified illuminated images permits both inspection of the surface morphology of the cervix and facilitates expert telemedicine quality assurance. Emphasizing sensitive criteria to avoid missing precancer/cancer, ~ 25% of women screen positive, reflecting partly the high HIV prevalence [17, 18]. As a research ‘add-on’ for this study, the nurses also took an additional triplicate set of images using a Samsung Galaxy J8™ smartphone camera. They also collected a cervical swab that was sent for subsequent HPV testing using the BD Onclarity™ assay system installed in Lusaka at a major referral hospital.

Expert gynecologic pathology review was available. Cases of cervical precancer/cancer were defined clinically as women having histologic CIN2, CIN3, or cancer. Glandular neoplasia was uncommon and grouped with the corresponding severity of squamous diagnoses (AIS with CIN3, ADC with SCC). Controls were women with completely visible squamocolumnar junctions (Type 1 or 2 Transformation Zones) whose visual screen was judged to be normal and not requiring referral for biopsy, combined with those that were referred but had histologic findings < CIN2.

### HPV testing

The results of the HPV testing performed in Lusaka were obtained by Onclarity batch testing for research purposes only, and unconnected to clinical management. Onclarity provides HPV typing that can approximate the type groups ranked in order of carcinogenicity [19]. Specifically, the assay yields results individually for HPV 16, 18, 31, 45, 51, and 52, but combines 33/58, 56/59/66, and 35/39/68. For the purposes of this research the results were further grouped based on established risk of cancer in a hierarchical classification as HPV16, else HPV18/45, else HPV 31/33/52/58, else HPV 35/51/56/59/66/68. Of note, the inclusion of HPV 35 in the lowest risk group is now known to be an error (among individuals of African heritage, it properly belongs with the other HPV 16-related types in the HPV 31 group) [20], and the incorrect inclusion of HPV 66 as carcinogenic is another acknowledged limitation of this assay [21], leading to some false positives.

### Automated visual evaluation (AVE) algorithm

The AVE algorithm was pre-trained on the NCI cervical image bank that contains more than 150,000 images taken with Cerviscopes (35 mm film images called Cervigrams, subsequently digitized) or DSLR camera images taken by beam splitting of Zeiss colposcope images [13]. The reader is referred elsewhere for detailed description of the logic, training, and initial validation of this deep-learning algorithm [12–15]. As noted above, the algorithm yields an ordered three-level classification of severity ("likely precancer/cancer", "indeterminate", or "normal" appearance). Its performance has been validated on internal "hold-back" test sets but, prior to this presentation, had not yet been validated in combination with HPV genotyping on an external dataset using a different image device in a distinct screening population.

### Treatment and histologic diagnoses

An important aspect of the Zambian screening program is expert treatment of screen-positive women [16]. If visually assessed lesions meet the WHO criteria for ablation by cryotherapy or thermal ablation of the transformation zone, that treatment is performed [22]. For the purposes of this research, women underwent biopsy prior to ablation to detail underlying pathology. If more extensive treatment was needed, either LLETZ was performed or punch biopsies were taken to exclude invasion as guided by clinical assessment or/and expert review of digital cervigrams.

The case and control histologic diagnoses in this study were based therefore on punch biopsies or LLETZ specimens, evaluated by an expert pathologist. As stated above, women that screened negative were also included as controls despite having no biopsy (as were those with negative digital cervicography/biopsy) given the very sensitive threshold for VIA positivity, high rates of referral, and the substantial expertise of the examining nurses.

### Data analysis

**Fig. 2:**
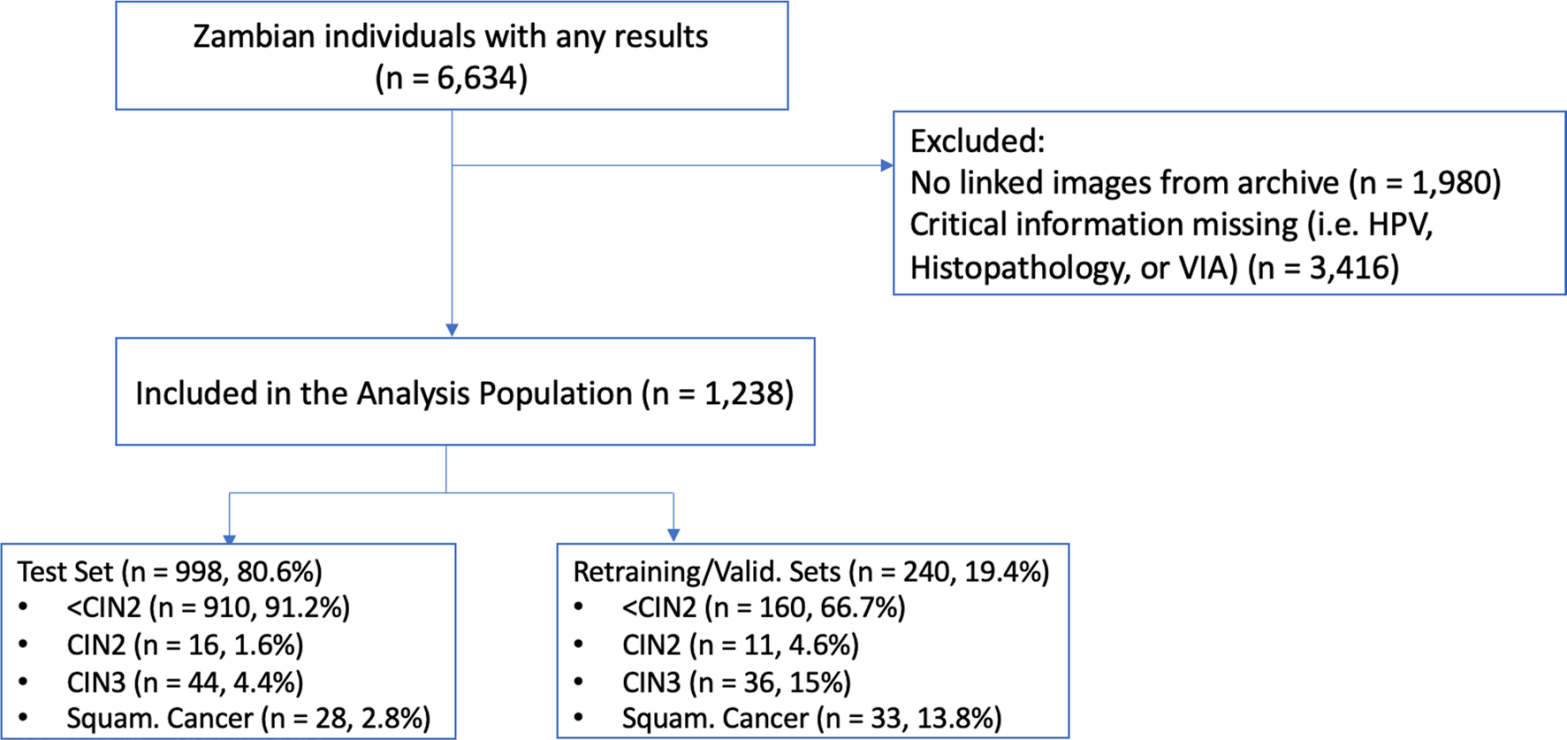
Consort diagram of Zambia dataset

The population diagram for the study is shown in Fig. 2. The associations of HPV type group and AVE classification with histologic outcome were visualized for all women having all three variables (Additional file 1: Fig. S1).

The data analysis included the following: First, we tested transfer learning of the candidate AVE algorithm for immediate use without modification on the J8 images. The J8 image type was a kind not previously included in AVE training. We postulated that portability might require retraining of the AVE algorithm to permit familiarity with the new image type. A small subset of images from women in the Zambian screening clinic was used for retraining, and contained 80 individuals with each classification (precancer/cancer, indeterminate, normal). The retraining images were added incrementally (20, then 40, then 60, then 80) to the NCI core collection to consider incrementally how many of the previously unfamiliar kind of images were needed to transfer the algorithm successfully; 40 was the chosen number achieving reasonable performance (Additional file 1: Table S1).

Once AVE was trained to analyze the Zambian J8 images, we assessed repeatability of the AVE results obtained from the three replicate images of the same individual captured by the J8 smartphone camera. Repeatability was assessed as an ordinal 3 × 3 table since the output was three ordinal classes of increasing severity: normal, indeterminate (HPV-positive patients with some equivocal/borderline/look-alike cervical changes), and precancer/cancer. In assessing reproducibility, the percent of the individuals that were extremely misclassified on replicates was of special interest (i.e., normal images classified as precancer/cancer, or vice versa).

Accuracy of a test is typically judged to be the correct identification of cases and non-cases, generally assessed in a 2 × 2 table by sensitivity, specificity, and their tradeoff (area under the receiver operating curve, or AUC). However, in this version of AVE, a large "gray zone" of indeterminate results was established between precancer/cancer and normal. Thus, the analysis assessed a 3 × 3 matrix (which shows the three diagnostic truth classes as one dimension and three-level test classification as the other). The worst inaccuracies, i.e., the percent of extreme errors, were again of special interest (precancer/cancer called normal, or vice versa).

## Results

Shown in Table 1 are the general characteristics of the Zambian screening population. The variables including HIV, HPV genotyping, histopathology, VIA classification, and ground truth classes for AVE algorithm are presented. Of note, a high percentage, 35%, of the women in the total analysis population (test set in Table 1) were HIV-positive.

**Table 1:** Summary of Zambia screening population (test set n = 998 and retraining/validation set n = 240)

	N (test set)	%	N (retraining/validation set)	%
HIV				
HIV–	620	62	116	48
HIV+	354	35	122	51
Unknown	24	2	2	0.8
HPV				
HPV 16+	88	9	54	23
HPV 18/45+	85	9	26	11
HPV 31/33/52/58+	126	13	34	14
HPV 35/39/51/56/59/66+	62	6	22	9
HPV HR negative	637	64	104	43
Histopathology result				
No histology	759	76	128	53
Negative	88	9	17	7
CIN1	63	6	15	6
CIN2	16	2	11	5
CIN3	44	4	36	15
Squamous cancer	28	3	33	14
VIA				
Negative	750	75	122	51
Positive	207	21	85	35
Cancer	41	4	33	14
AVE ground truth				
Normal	550	55	80	33
Indeterminate	365	37	80	33
Precancer+	83	8	80	33

### Non-portability of AVE algorithm to new image type

The initial "transfer learning" application of the pre-trained AVE algorithm to the Zambian J8 image set generated very poor performance, with nearly random discrimination of the reference diagnostic classes (Additional file 1: Table S1).

Retraining was required, i.e., adding case/control J8 images from the Zambia dataset into our original training/validation sets. We tried including 17 + 3, 35 + 5, 52 + 8, 70 + 10 (training + validation) individuals’ data from each ground truth class (for example, we randomly selected 40 individuals with ground truth of normal, indeterminate, and precancer/cancer each 35 to be added into our training set and five to be added into our validation set, which is used during retraining at various checkpoints to monitor the progress of the retraining). Except for the experiment with only 17 + 3 individuals’ data addition, all other retrained algorithms performed well (Additional file 1: Table S1). For the rest of this section, we will present validation results of the AVE algorithm retrained with additional 35 + 5 (training + validation images) individuals’ data from each diagnostic class.

### Repeatability

**Fig. 3:**
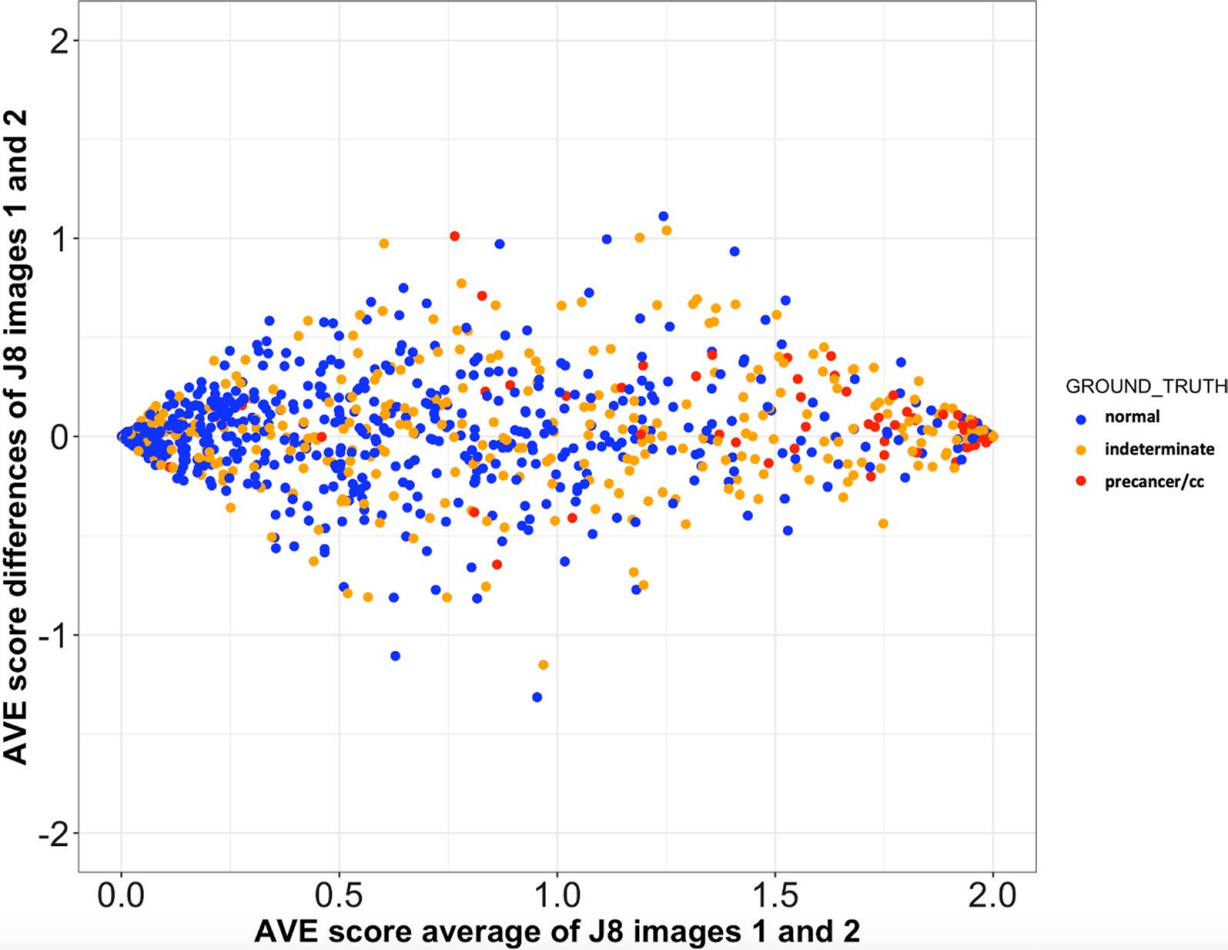
Bland–Altman plot—assessing the repeatability of AVE scores obtained from 2 different J8 images captured from the same patient at the same screening visit. It displays the repeatability of AVE scores obtained from 2 different J8 images captured from the same patient at the same visit by Bland–Altman plot. The x-axis shows the average of 2 AVE scores obtained from 2 different J8 images while the y-axis shows the difference of these scores. Continuous AVE score is obtained as the summation of class label (0, 1, 2) multiplied by its corresponding class AVE prediction. Each point in the plot is colored according to its ground truth. Blue points represent ground truth normal patients, yellows are indeterminate cases, and reds are confirmed CIN2+ cases. Under perfect repeatability, score differences are expected to be zero; therefore, in an ideal situation, all of the points on the graph are expected to be lying on the y = 0 line (horizontal line passing through 0). However, in our situation points vary around this horizontal line, and the variability is highest at the middle (where x = 1). This means that the variability in score differences is dependent on score averages. The variability is smallest at each end 0 (corresponding to normal) and 2 (corresponding to precancer/cc), and is highest at the middle which means there is low repeatability at indeterminate class compared to definite normal and precancer/cancer classes

Table 2 and Fig. 3 present repeatability results of the retrained algorithm as measured in the test set. Each individual in this dataset had on average three images captured by Samsung J8. Table 2 compares the AVE result from the first two J8 images captured from the same patient at the same visit, as a 3 × 3 ordinal matrix with AVE predictions on the first J8 image as one dimension and the second image on the other. Of the individuals in the test set, 79% had the same AVE test result on both images with weighted kappa score 0.72 (95% CI 0.68–0.76). The other pairwise comparisons (first image versus third, second versus third) generated comparable results.

**Table 2:** Repeatability of AVE algorithm results obtained from 2 different J8 images captured from the same patient at the same visit

J8 image 1	J8 image 2			
	Normal (n = 441)	Indeterminate (n = 261)	Precancer+ (n = 218)	No Result*
Normal (n = 459)				
n	377	67	15	2
% of total	41%	7.3%	1.6%	
Indeterminate (n = 264)				
n	56	175	33	3
% of total	6.1%	19%	3.6%	
Precancer+ (n = 197)				
n	8	19	170	6
% of total	0.9%	2.1%	19%	
No result*				
n	0	0	0	67

It shows agreement between two J8 images obtained from the same patients at the same visit. Overall, there is 79% agreement between the AVE classification results obtained from two J8 images. The weighted kappa score between these 2 images is 0.72 (95% CI 0.68–0.76)

*67 individuals have no J8 images and 11 (2 + 3 + 6) individuals only have 1 J8 image (no replicates). These 67 individuals have images captured by other camera types

Figure 3 displays the difference between the two replicate image scores on the y-axis plotted against the average of them on the x-axis (a Bland–Altman plot). Continuous scores were obtained by multiplying predicted class probabilities given by the AVE algorithm with their corresponding class labels (0: normal, 1: indeterminate, 2: precancer/cancer). Most of the normal class images (represented by blue dots) are clustered on the left end of the graph with only small variability on the y-axis, meaning most of the normal images were repeatedly estimated as normal across the replicate images. The same holds for the precancer/cancer cases (red dots) as well; however, the variability of the continuous score (i.e., larger differences between replicates) increases somewhat at the middle (indeterminate class images). In other words, indeterminate images generated slightly more variable AVE results.

### Accuracy

The AVE classification trended strongly toward more severe classification linked to the severity of the histologic diagnosis (Additional file 1: Fig. 1). Table 3 displays accuracy of the algorithm results among HPV risk groups, compared with the actual histologic results. Both tests showed strong associations with case-indeterminate-control status.

**Table 3:** Results by histology of HPV then AVE from J8 images, pretrained algorithm retrained with 35 + 5 patients’ data added to each class from Zambia screening population

HPV	AVE	Histology status						Total
		< CIN2		CIN2		CIN3+		
		n	%	n	%	n	%	n
HPV 16+	Precancer/cancer	12	1.4	0	0.0	21	39.6	33
	Indeterminate	11	1.3	2	14.3	1	1.9	14
	Normal	21	2.4	2	14.3	4	7.5	27
	No result*	4	–	1	–	9	–	14
	Subtotal	48	5.1	5	28.6	35	49.1	88
HPV 18/45+	Precancer/cancer	10	1.2	1	7.1	6	11.3	17
	Indeterminate	13	1.5	0	0.0	3	5.7	16
	Normal	23	2.7	0	0.0	0	0.0	23
	No result*	1	–	0	–	5	–	6
	Subtotal	47	5.3	1	7.1	14	17.0	62
HPV 31/33/52/58+	Precancer/cancer	30	3.5	3	21.4	9	17.0	42
	Indeterminate	32	3.7	1	7.1	1	1.9	34
	Normal	43	5.0	0	0.0	0	0.0	43
	No result*	5	–	1	–	1	–	7
	Subtotal	110	12.2	5	28.6	11	18.9	126
HPV other HR+	Precancer/cancer	14	1.6	0	0.0	3	5.7	17
	Indeterminate	24	2.8	0	0.0	2	3.8	26
	Normal	35	4.1	0	0.0	0	0.0	35
	No result*	3	–	0	–	4	–	7
	Subtotal	76	8.4	0	0.0	9	9.4	85
HPV High risk−	Precancer/cancer	90	10.4	2	14.3	2	3.8	94
	Indeterminate	176	20.4	0	0.0	1	1.9	177
	Normal	330	38.2	3	21.4	0	0.0	333
	No result*	33	–	0	–	0	–	33
	Subtotal	629	69.0	5	35.7	3	5.7	637
Total		910	100	16	100	72	100	998

*67 individuals are missing J8 image so they do not have AVE test result. These 67 individuals have images captured by other camera types

Percentages exclude individuals with missing images

### Risk stratification

**Fig. 4:**
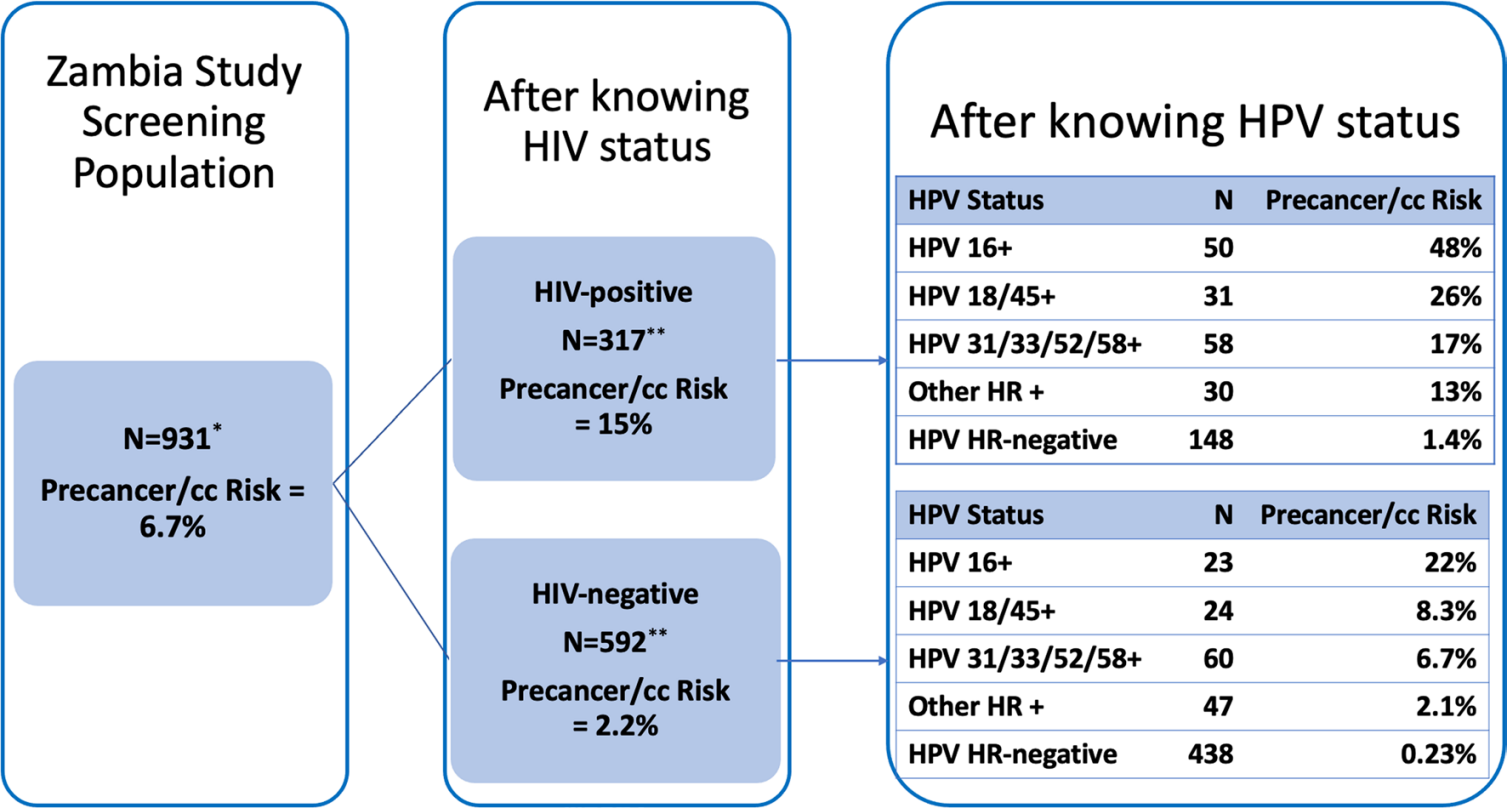
Step by step precancer/cancer stratification: low prevalence Zambia study population, after knowing HIV status, after knowing HPV status. This figure explains step by step risk discrimination in a population after knowing each screening test result. In total, there are 931 patients screened in this study in Zambia (67 excluded due to no J8 image). After testing for HIV, precancer/cc risk of HIV+ patients increases to 15% while the risk decreases to 2.2% for HIV-negative patients. After HIV, if the patients get tested for HPV genotype, we can observe even further risk discrimination. A 15% precancer/cc risk of HIV-positive patients increases to 48% if they are positive for HPV type 16. Similarly, the risk decreases from 15 to 1.4% if the patients are HPV HR-negative. For HIV-negative patients, the precancer/cc risk increases from 2.2 to 22% if they are positive for HPV type 16. Similarly, if HIV-negative patients are negative for any HPV HR types then their precancer/cc risk decreases to 0.23%. In the above figure, the number of patients (N) observed in each category and their precancer/cc risk are displayed separately for each category. *67 individuals have no J8 images, they have images captured by other camera types. **37 of HIV+ individuals and 28 of HIV− individuals, and 2 of the HIV missing individuals do not have any J8 images (which add up to 67 from the previous step), so they are not included in this analysis. 22 individuals have missing HIV result (317 HIV+, 592 HIV − , and 22 missing HIV will add up to the previous step, screening population)

**Fig. 5:**
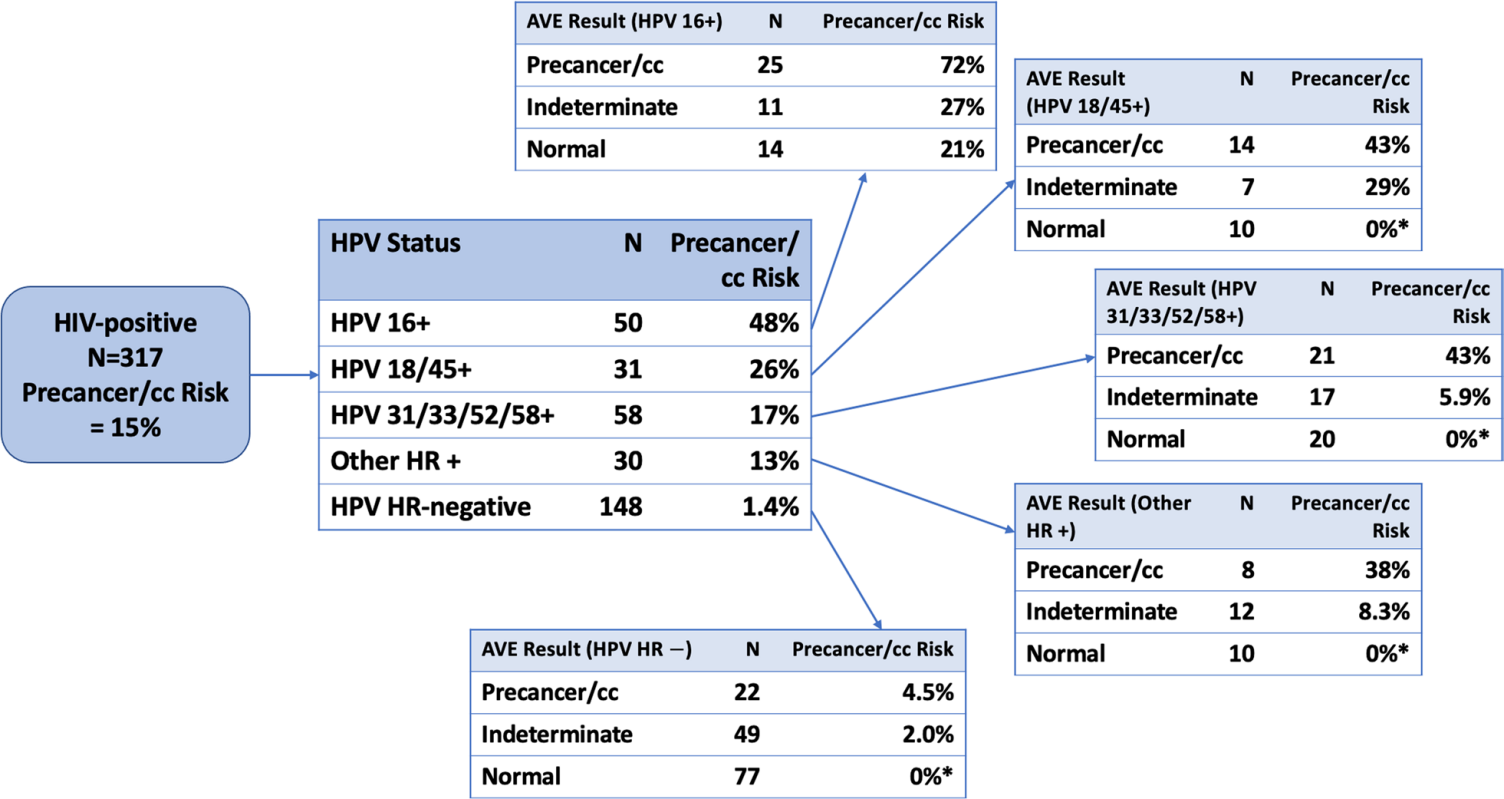
Precancer risk stratification among low-prevalence HIV-positive Zambia study population by HPV and AVE combined results. This figure is an extension of Fig. 4 extending the risk discrimination to demonstrate the intended use case of PAVE, triaging HPV-positive individuals with HPV genotyping and AVE. The population is first tested for HIV, and this figure shows the risk discrimination among HIV-positive patients first tested with HPV and then with AVE. As demonstrated in previous images, 15% precancer/cc risk of HIV-positive patients will vary between 48 and 1.4% (HPV 16+ and HPV HR-negative, respectively) after being tested for HPV genotype. If we apply AVE test after HPV genotype, we can observe even finer risk discrimination such that 48% precancer/cc risk of HIV-positive and HPV 16+ patients will increase to 72% for AVE result precancer/cc and decrease to 21% for AVE result normal. The highest risk group is HPV 16+ and AVE precancer/cc, followed by other HPV-positive groups and AVE precancer/cc. The lowest risk groups are HPV HR-negative and AVE normal/indeterminate with 0–2.0% precancer/cc risk. 37 of HIV+ individuals do not have any J8 images (their images were captured by other camera types), so they are not included in this analysis. *No cases observed in these categories

As shown in Figs. 4 and 5, at each step in the screening/triage strategy, we estimated pre- and post-test chance of having precancer/cancer. We considered sequentially the data available on the different variables, to simulate independent performance. Both kinds of triage tests (AVE and HPV type) were linked strongly with histologic diagnoses.

Figure 5 demonstrates the use case of HPV and AVE together. The overall precancer/cancer risk of the population was 6.7%. We divided by HIV status. HIV-negative women had very low risk of CIN2 or worse, with so few cases that fine stratification by both HPV type group and AVE was not feasible (5 CIN2+ among HPV 16+, 7 CIN2+ among other hrHPV+, and 1 CIN2+ among hrHPV-). The precancer/cancer risk of HIV-positive patients varied between 48 and 1.4% after testing for HPV status (HPV 16+ and HPV HR-negative, respectively). When we added the AVE test after HPV, we observed even finer risk discrimination such that 48% precancer/cancer risk of HIV-positive and HPV 16+ patients increased to 72% for AVE result precancer/cancer and decreased to 27% and 21% for AVE result indeterminate and normal, respectively.

Additional file 1: Figure S2 presents the rank order of the individuals according to their predicted chance of having cervical precancer/cancer. The figure demonstrates the population (x-axis) ranked based on the HPV-AVE algorithm and compares what percent of the observed precancer/cancers (y-axis) in this population could be detected if a certain percent of the high-risk population is referred for management. In this figure, the HIV-positive population is demonstrated because there are more precancer/cancer outcomes permitting stratification of risk. In this example, one possible threshold or cutpoint for managing patients could be drawn at risks equal or greater than that observed for women that are HPV16+ and normal AVE. At this cutpoint, 92% of the expected precancers would be detected and treated. To achieve this sensitivity, 31% of the HIV-positive population would be referred for management.

## Discussion

The data support that a combination of HPV typing and automated visual evaluation (AVE) could accurately distinguish women at different risks of cervical precancer/cancer. Current cervical cancer prevention guidelines in the US and Canada employ the principles of risk-based management [23, 24]. Applying risk-based management in resource-limited settings is paramount as resources are concentrated on patients at highest risk of cancer, and the harms of overtreatment are avoided in those at low risk [3]. Using HPV testing with genotyping and AVE for risk-based management would not require new scientific discovery, but the real-life challenge of establishing the strategy in a lower-resource setting like the Zambian public sector is of paramount importance.

As one possible strategy, the screening process could start with collecting self-sampled vaginal swabs from the general screening population [25] and evaluating of these samples by a sensitive target-amplification test like the new ScreenFire HPV test [26]. (under review). This HPV test will give genotyping results for the high-risk HPV-positive patients in the 4 hierarchical channels, which are HPV 16, else HPV 18 or 45, else HPV 31, 33, 35, 52, 58, else HPV 39, 51, 56, 59, 68. After obtaining the HPV test result, patients with high-risk HPV-positive could have an image captured by a dedicated hand-held camera (i.e., cell phone camera, digital camera, or other local choice). The cervix image would be evaluated by the AVE algorithm to give one of the three test results: normal cervix, indeterminate (neither completely normal nor a precancer/cancer), or precancer/cancer. This deep-learning based screening test, AVE, would be an assistive technology to guide clinicians in these settings, reducing the number of unnecessary referrals for treatment or workup.

The 12-level screening table would indicate each woman's chance of having precancer/cancer, and could be used by the decision-makers in the local public health and clinical authorities to create risk-action thresholds. In other words, those in charge of each setting could decide based on resources and risk tolerances how to convert the risks into actions, similar to how risks are used in current US and Canadian guidelines [23, 24].

The strengths of this study include a large dataset of images from women with and without HIV, collection of HPV testing with genotyping, availability of multiple (triplicate) cervical images, and cervical disease outcomes on all patients. The nurses performing the standard of care VIA screening aided by digital camera imaging are highly trained, and have individually performed thousands of screenings, and often act as master-trainers for colleagues across the country and region. Adding to the fact that they also have an internal quality assurance program, the quality of VIA results in this study is expected to be much higher than is reported in most studies worldwide [17, 27].

The limitations of this convenience dataset include inclusion only of women with complete data by the study end, the assumption that VIA-negative patients did not have precancer, and the potential limitations of the Onclarity HPV assay (e.g., the inclusion of a lower-risk type HPV66, grouping of HPV35 with lower risk HPV types that is inconsistent with true precancer/cancer risk in an African population [20]), and the low numbers of precancer/cancer in the HIV-negative population precluding detailed analysis. Finally, the AVE algorithm was not run on a smartphone camera itself (was run offline using high intensive graphic cards/chipsets on computers in the lab). Adapting and miniaturizing high performing AVE algorithms onto lower-cost devices is a major technical and operational requirement for this technology to be transformed into a near-patient/point-of-care clinical application [28].

AVE shows promise as an assistive technology when performing screen-triage-and treat strategies [13–15]. However, this and other studies indicate that AVE cannot be applied “out of the box” to new patient populations or used with different image capture devices than those used to train the original algorithm [12]. Unless setting-specific images are provided to retrain the algorithm, it will fail to distinguish precancer/cancer at a rate much higher than chance alone [12]. This study suggests that approximately 40 images (35 training + 5 validation) of each class (normal, indeterminate, precancer+) could retrain the algorithm to function in a new setting. Obtaining these data would require dedicated protocols to obtain images and pathology specimens, which in turn could require screening of several hundred to several thousand women in each new setting. Finally, there is a theoretical potential for AVE-assisted VIA as a primary visual screening approach that could replace VIA as—at least—a non-inferior alternative to HPV-based primary screening, especially in settings of great burden where HPV remains unavailable or unaffordable. However, the formal evaluation of this strategy requires rigorously conducted clinical effectiveness, cost effectiveness, and implementation science studies.

## Conclusion

Given advanced understanding of cervical cancer etiology and pathogenesis, and effective prevention methods, the critical measure of success/failure is whether we actually save lives and reduce suffering from cervical cancer [10]. Cervical cancer is already and increasingly linked to deeply inequitable distribution of prevention efforts [29–31]. The vital need is for immediate prioritization of prevention efforts where they are lacking. The HPV typing and AVE visual triage approach has promise but will need evaluation in clinical effectiveness and implementation studies to determine if it is indeed a feasible and affordable real option in settings of great need like Zambia.

### Supplementary Information

**Additional file 1. Supplemental Table 1:** Portability Analysis for J8 images (Comparison of models retained with different-sized Zambia data). **Supplemental Figure 1**: Assessing AVE predictions under each histologic and HPV genotype result. **Supplemental Figure 2**: Concentration curve for HIV-positive study population shows what percent of the high-risk study population needs to be referred for management to detect a certain percentage of expected precancers in that study population.

## Data Availability

Data share requests should be referred to Dr. Groesbeck Parham. Questions regarding the output of AI algorithm should be referred to Dr. Mark Schiffman.
