# Supplementary material for: Validation in Zambia of a cervical screening strategy including HPV genotyping and artificial intelligence (AI)-based automated visual evaluation
Source: Infect Agent Cancer. 2023 Oct 16;18:61. doi: 10.1186/s13027-023-00536-5 (PMC10580629; doi:10.1186/s13027-023-00536-5)
Supplement: Supplementary file 1 — Additional file 1. Supplemental Table 1: Portability Analysis for J8 images (Comparison of models retained with different-sized Zambia data). Supplemental Figure 1: Assessing AVE predictions under each histologic and HPV genotype result. Supplemental Figure 2: Concentration curve for HIV-positive study population shows what percent of the high-risk study population needs to be referred for management to detect a certain percentage of expected precancers in that study population. [file 13027_2023_536_MOESM1_ESM.docx]

**SUPPLEMENTAL MATERIAL**

**Supplemental Table 1:** Portability Analysis for J8 images (Comparison of models retained with different-sized Zambia data)

|  |  | **Size of retraining/validation sets** | | | | | | | | | | | | | | |
| --- | --- | --- | --- | --- | --- | --- | --- | --- | --- | --- | --- | --- | --- | --- | --- | --- |
|  |  | **0/0** | | | **17/3** | | | **35/5** | | | **52/8** | | | **70/10** | | |
| **Camera** | **Cut Point for Case Status** | **Sens.** | **Spec.** | **AUC** | **Sens.** | **Spec.** | **AUC** | **Sens.** | **Spec.** | **AUC** | **Sens.** | **Spec.** | **AUC** | **Sens.** | **Spec.** | **AUC** |
| J8 image 1 | Precancer/cc | 83.9% | 39.6% | 0.62 | 64.5% | 85.4% | 0.77 | 72.6% | 81.8% | 0.80 | 74.2% | 84.8% | 0.84 | 77.4% | 86.3% | 0.86 |
|  | ≥Indeterminate | 100.0% | 0.0% |  | 100.0% | 12.0% |  | 90.3% | 52.4% |  | 96.8% | 45.5% |  | 93.5% | 60.0% |  |
| J8 image 2 | Precancer/cc | 93.4% | 36.8% | 0.65 | 77.0% | 84.6% | 0.82 | 82.0% | 80.4% | 0.83 | 83.6% | 82.5% | 0.86 | 78.7% | 85.2% | 0.85 |
|  | ≥Indeterminate | 100.0% | 0.0% |  | 98.4% | 11.5% |  | 91.8% | 50.8% |  | 98.4% | 46.7% |  | 91.8% | 56.8% |  |
| J8 image 3 | Precancer/cc | 85.7% | 36.7% | 0.63 | 71.4% | 85.1% | 0.80 | 80.4% | 81.2% | 0.82 | 71.4% | 84.5% | 0.82 | 78.6% | 85.0% | 0.85 |
|  | ≥Indeterminate | 98.2% | 0.0% |  | 100.0% | 11.9% |  | 89.3% | 55.4% |  | 94.6% | 46.9% |  | 91.1% | 60.6% |  |

**Supplemental Figure 1:** Assessing AVE predictions under each histologic and HPV genotype result.

**
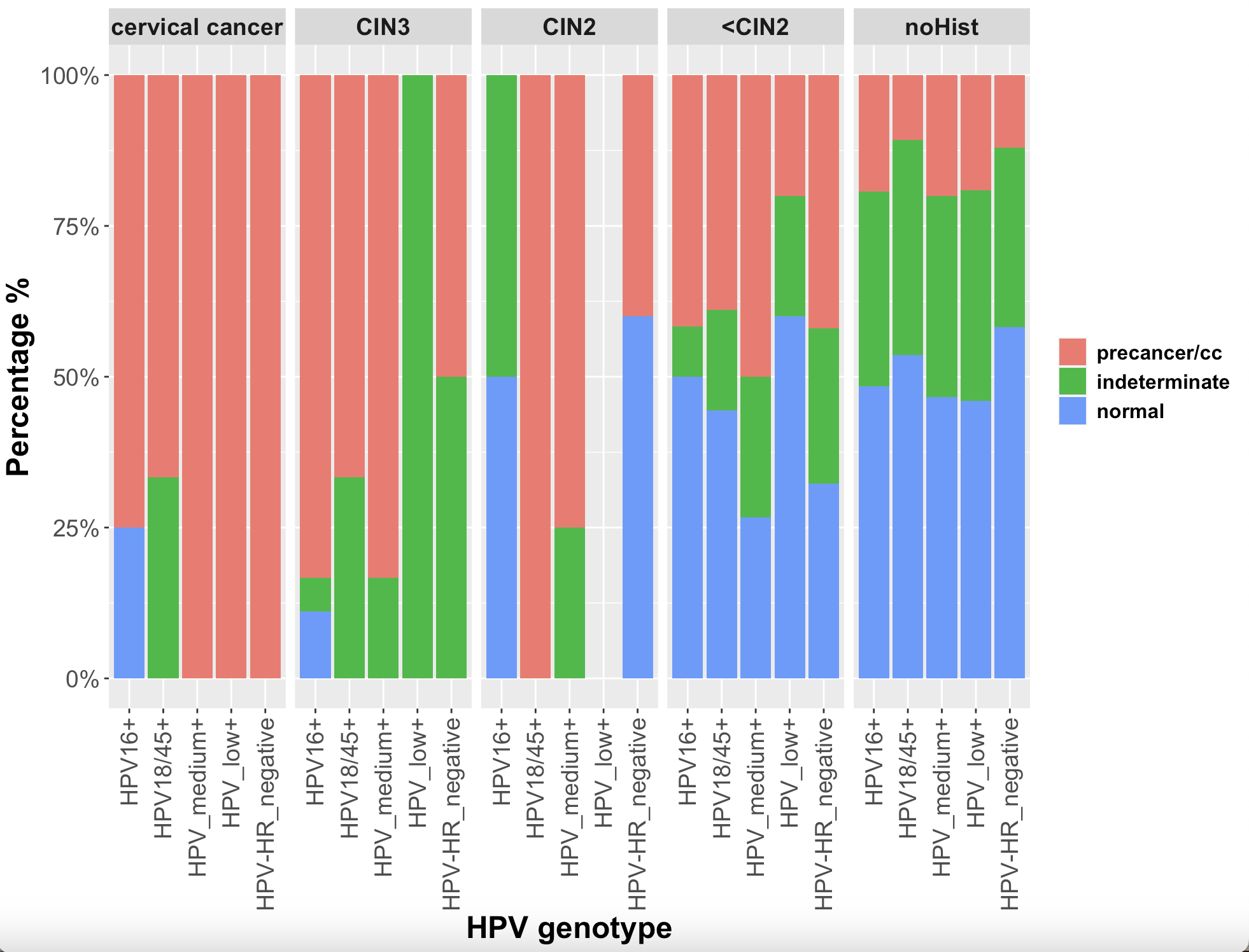
**

| In the above figure, we demonstrate the AVE class predictions under each histology and HPV genotype groups visualizing Table 3. As the histology category severity decreases, the normal class prediction of AVE model increases as expected. |
| --- |

**Supplemental Figure 2:** Concentration curve for HIV-positive study population shows what percent of the high-risk study population needs to be referred for management to detect a certain percentage of expected precancers in that study population.


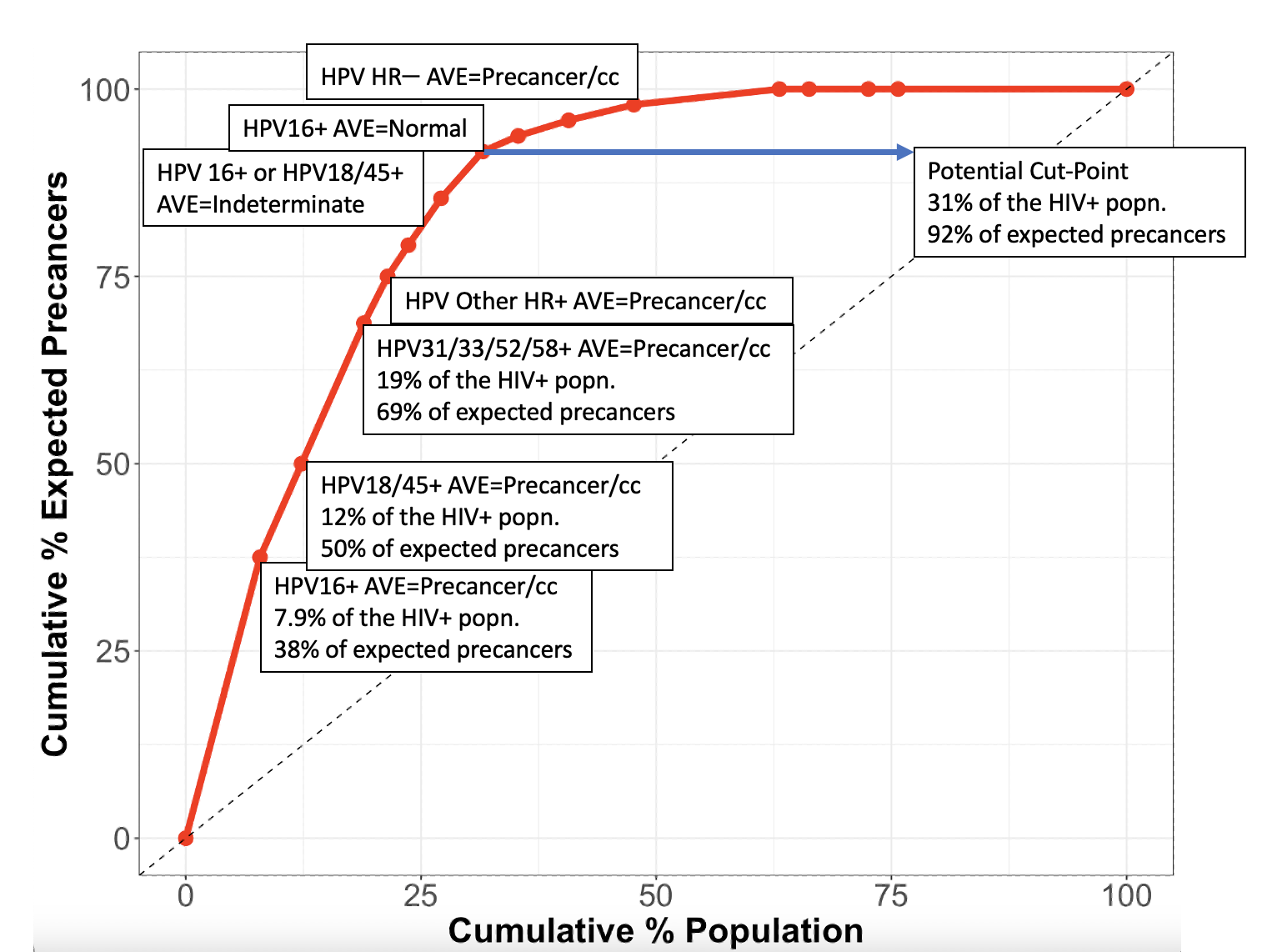


| This figure is called concentration curve and demonstrates a population ranked from highest risk to lowest risk of precancer/cc risk and expected precancers in this population. In this figure, we are showing HIV-positive population in Zambia. We assume that the HIV-positive sample we have is a good representative of the HIV-positive population in Zambia. This curve shows risk discrimination achieved between high- and low-risk patients (risks according to PAVE result) by looking at the expected precancers or cancers at each risk group. For instance, the highest risk group with 72% precancer/cc risk (shown in Figure 5) is HPV 16+ and AVE category precancer/cc. Patients in this category are 7.9% of the whole HIV-positive population and by only looking at this group 38% of the expected precancers/cancers in this population can be detected. If we combine the highest risk groups (HPV-positive, any type, and AVE category precancer/cc), they are 21% of the HIV-positive population and 75% of the expected precancers can be detected by looking at this group. If a cut-point for management is sought in this population, it will be ideal to put it at HPV 16+ and AVE normal result (blue arrow). In that situation, 31% of the HIV-positive population will be managed and 92% of the expected precancers/cancers in this population will be eliminated. |
| --- |
